# Salivary fluoride concentration following toothbrushing with and without rinsing: a randomised controlled trial

**DOI:** 10.1186/s12903-022-02086-5

**Published:** 2022-03-03

**Authors:** Marwah M. Albahrani, Asma Alyahya, Muawia A. Qudeimat, K. Jack Toumba

**Affiliations:** 1 Department of Paediatric Dentistry, Farwanyia Speciality Dental Centre, Farwanyia, Kuwait; 2 Department of Developmental and Preventive Sciences, Kuwait University, Jabriyah, Kuwait; 3 Department of Paediatric Dentistry, The University of Leeds School of Dentistry, Clarendon Way, Leeds, LS2 9JT West Yorkshire UK; 4 Faculty of Dentistry, Kuwait University, PO Box: 24923, Safat, 13110 Kuwait

**Keywords:** Fluoride toothpaste, Rinsing, Controlled clinical trial, Adults

## Abstract

**Background:**

Caries prevalence has declined significantly since the introduction of fluoridated toothpaste. There have been several developments regarding specific active fluoride ingredients but not enough evidence to support one over the other. The purpose of this double-blind randomized controlled trial was to compare salivary fluoride concentrations of different fluoride formulations in the form of toothpaste with and without post-brushing water rinsing in adults.

**Methods:**

The study included 120 participants who were randomly assigned to one of 12 groups (10 participants/group). The toothpaste formulas investigated included (1) fluoride-free (0 ppmF); (2) sodium fluoride (1450 ppmF); (3) sodium monofluorophosphate (1450 ppmF); (4) sodium fluoride and monofluorophosphate combined (1450 ppmF); (5) stannous fluoride and sodium fluoride combined (1450 ppmF); and (6) amine fluoride (1400 ppmF). Block randomisation was used to assign each participant to one of the 12 groups. Participants brushed with 1.0 g of one of the six different toothpaste formulations either with or without post-brushing water rinsing. Saliva was collected at six different times (baseline and at 1, 15, 30, 60, and 90 min/s post-brushing). Samples were analysed using a fluoride ion-specific sensitive electrode connected to an ion analyser.

**Results:**

The demographic characteristics of the participants were not significantly different among the groups (*P* > 0.05). Time, toothpaste formulation, and post-brushing rinsing routines had significant effects on saliva fluoride retention (*P* < 0.05). Amine fluoride-containing toothpaste was the only formula that showed statistically significantly higher concentrations of salivary fluoride at 90 min in both the rinsing and non-rinsing groups. Sodium monofluorophosphate toothpaste did not result in a significant difference compared to the control group at any time point, in both rinsing and non-rinsing groups.

**Conclusions:**

Based on the results from this study, no rinsing after toothbrushing in adults can be recommended when sodium monofluorophosphate containing toothpaste formula is used. It also concludes that amine fluoride resulted in a significantly higher saliva fluoride concentration at 90 min in both the rinsing and non-rinsing groups compared to other fluoride toothpaste formulations.

*Registry*: Protocol Registration and Results System (ClinicalTrials.gov).

*Clinical trial registration number*: NCT02740803 (15/04/2016).

## Introduction

Dental caries is the most prevalent chronic disease worldwide. One of the widely accepted cost-effective methods of caries prevention is toothbrushing with fluoridated toothpaste. This method of delivering high-dose fluoride over a low-frequency regimen has proven its effectiveness in reducing the incidence of caries [1–4]. Systematic reviews and meta-analyses have confirmed the benefit of 1000–1500 ppm F-containing toothpaste to achieve caries prevention [5–7].

Fluoride-containing toothpaste is available in various chemical compositions and formulations. Amine fluoride (AmF), sodium fluoride (NaF), sodium monofluorophosphate (Na_2_FPO_3_), and stannous fluoride (SnF_2_) are the main active fluoride ingredients available in toothpastes. Clinical trials suggested the superiority of NaF composition over SnF_2_ and Na_2_FPO_3_ favouring NaF containing toothpaste [8–10]. Others suggested that AmF toothpaste resulted in higher salivary fluoride concentrations and therefore, marked remineralization of caries compared to NaF and Na_2_FPO_3_ containing toothpastes [11–14].

Post-brushing rinsing is an area of interest for many researchers exploring the anti-caries effectiveness of fluoride toothpastes. Post-brushing rinsing and the availability and rate of fluoride clearance from the oral cavity were investigated in several studies [12, 13, 15, 16]. Rinsing post-brushing was found to result in significantly lower salivary fluoride concentrations compared to non-rinsing groups [12, 13, 16, 17]. However, it has been concluded that there are no high-quality studies to support the comparative effectiveness of one active fluoride toothpaste formula over the other with or without post-brushing mouth rinsing [18]. Many previous clinical trials that investigated this subject had methodological limitations, including (1) inclusion and exclusion criteria were not clearly described [9, 11–13, 16], (2) sample size calculations not performed [9, 11–13, 16, 17], (3) lack of randomisation [9, 11, 17], and (4) examiners were not blinded to the intervention group [11, 17]. Therefore, the aim of this study was to conduct a randomised double-blind clinical trial to compare the salivary fluoride concentrations following toothbrushing with different fluoride toothpaste formulations with and without post-brushing water rinsing. The null hypotheses were: 1. Toothpaste formulations with similar fluoride concentrations have no significant differences in terms of salivary concentrations of fluoride when measured at different time intervals; 2. There are no significant differences between post-brushing rinsing and non-rinsing regarding salivary fluoride concentrations for the tested toothpaste formulations.

## Materials and methods

This was a double-blind randomized controlled trial (RCT). Ethical approval was obtained from the Yorkshire and The Humber—Sheffield Research Ethics Committee (REC reference number 16/YH/0015). The trial protocol was registered with ClinicalTrials.gov (NCT02740803-15/04/2016).

Sample size calculations were performed using Power Analysis and Sample Size Software (version 11.0, NCSS Statistical Software, Kaysville, Utah, USA). The study aimed to test 12 groups; each group was tested at 6 different time intervals. For this study, the confidence intervals were set at 95%, with 100% power. Sample size calculations were performed using raw data from a previous study [13]. A sample of at least three participants was needed for each group to achieve significant differences. It was decided to increase the final number of participants to 10 participants in each group, giving a total number of at least 120 participants. A circular email with an invitation to participate in the study was sent along with the recruitment flyer, the information sheet, and the consent sheet to students across the University of Leeds every two months starting from September 2016 until April 2017. Written consent was obtained from each participant.

To be included in the study, participants had to: 1. be adults with American Society of Anaesthesiologists (ASA) grades I or II; and 2. have a resting salivary flow rate of 0.1 ml/minute or more. Participants were excluded if they were: 1. edentulous; 2. allergic to any of the materials used in the study; 3. incapable of fasting for four hours; 4. unable of retaining the toothpaste following brushing; or 5. if they had orthodontic braces.

Block randomisation was used to assign participation numbers (1–120) to the participants in the groups (1–12) [https://www.randomlists.com/team-generator]. A trained research dental assistant randomised the toothpaste formulations with and without rinsing to the groups (1–12) using the same website. Then, she concealed the toothpaste tubes and labelled them. The participants and the principal investigator were blinded to the toothpaste formula. In addition, the principal investigator (who carried out the statistical analysis) was blinded to the rinsing methods.

The clinical examination and the research trial were undertaken at the Dental Translational and Clinical Research Unit at the University of Leeds, UK. The World Health Organization (WHO) criteria for Decayed, Missing due to caries, and Filled Teeth in the permanent teeth (DMFT), and Decayed, Missing due to caries, and Filled Surfaces in the permanent teeth (DMFS) scoring were followed. Teeth were also visually examined for the presence or absence of supra-gingival calculus. No aiding tools were used to detect small traces of calculus or subgingival calculus. The interventions were to brush with one of the following six different toothpaste formulations:

1. Control group (fluoride-free toothpaste): Kingfisher Natural Toothpaste ® Fennel-fluoride free—100 ml (Kingfisher Toothpaste, Norwich, UK).
2. Sodium fluoride toothpaste (1450 ppmF): Colgate Total ® Original Care™—125 ml (Colgate-Palmolive, Surrey, UK).
3. Sodium monofluorophosphate (1450 ppmF): Colgate Sensitive ® Pro-Relief™ Extra strength—75 ml (Colgate-Palmolive, Surrey, UK).
4. Sodium fluoride (450 ppmF) and monofluorophosphate (1000 ppmF) combined: Colgate ® Cavity Protection™—75 ml (Colgate-Palmolive, Surrey, UK).
5. Stannous fluoride (1100 ppmF) and sodium fluoride (350 ppmF) combined: Oral-B ® Pro-Expert™—75 ml (Procter & Gamble, Yorkshire, UK).
6. Amine fluoride (1400 ppmF): Elmex ® Protezione Carie—75 ml (Colgate-GABA; Switzerland)

All participants were instructed to refrain from brushing their teeth on the day of sample collection (the last time they could brush their teeth was the night before), and fast for at least two hours before their appointment and throughout the entire appointment. On the day of the experiment, each participant was asked to drool (unstimulated saliva sample) into a 15 ml sterile tube for two minutes to determine the salivary flow rate and the suitability of the participant to be included in the study. This sample was also used to determine the salivary fluoride concentration at baseline (pre-brushing sample). Participants were then asked to brush with a pre-weighed toothpaste (1.0 g) of one of 6 different fluoride toothpaste formulations for two full minutes. A timer was used to record the start and finish times of the brushing.

Depending on which group they were in, participants were either asked to spit the excess toothpaste and not rinse their mouth for the entire appointment or to rinse their mouth immediately following toothbrushing with 10 ml of distilled water for five seconds. After brushing, unstimulated saliva samples were collected five times at the following time intervals: 1, 15, 30, 60, and 90 min(s). Each saliva sample was collected in a pre-labelled test tube with the aid of a single-use funnel. Each sample was collected over two minutes. The brushing, rinsing, and collection of the saliva samples were supervised by a research assistant.

Each saliva sample tube was labelled with the participant’s screening number, date of collection, and time interval. Saliva samples were preserved in the laboratory freezer (-18 degrees Celsius) until they were analysed. The total duration of freezing the saliva samples did not exceed three months. On the day of the analysis, saliva sample tubes were taken out of the freezer two hours before the analysis. Equal parts of saliva samples and low-level Total Ionic Strength Adjustment Buffering solution (TISAB II) with cyclohexylenedinitrilotetraacetate (CDTA) were mixed in a sterile test tube. Fluoride concentrations were measured using a calibrated ion-specific sensitive electrode (Orion™ Model 9609BNWP, Thermo Fisher Scientific, Cambridgeshire, UK) connected to an ion analyser. Calibration of the fluoride ion-selective combination electrode was performed prior to sample measurement. Manufacturer instructions were followed to perform direct calibration using 0.01, 0.1, 1.0, 10, 100, and 1000 ppm fresh standard fluoride solutions mixed with equal parts of low-level TISAB with CDTA. As per the manufacturer’s instructions, the resulting slope value for the calibration process should be between − 54.0 and − 60 mV when the standards are between 20–25 ˚C. Recalibration was performed every two hours as per the manufacturer’s instructions. Recalibration of the electrode was performed when the reading of the values of the fluoride standards had changed by 2%. After each measurement session, the samples were safely disposed of as per the local protocols of the University of Leeds laboratories.

### Statistical analysis

The primary outcome was measuring salivary fluoride concentrations at baseline and post-brushing at 1, 15, 30, 60, and 90 min with and without rinsing. Statistical analyses were performed using the Statistical Package for the Social Sciences (SPSS) software (version 23.0, IBM Corp., Armonk, NY, USA). Prior to data analysis of salivary fluoride concentrations, missing data were replaced by multiple imputations. Before the replacement of missing data, pattern analysis was performed to investigate whether the missing data followed a certain pattern or a random arrangement. The predictor effects were considered to be statistically significant at ≤ 5% level.

Mauchly’s sphericity test was used to validate the repeated measures analysis of variance (ANOVA). Two-way mixed ANOVA with Tukey’s post-hoc test and Bonferroni correction were used for the data analysis of fluoride concentrations within the different groups at the different time intervals and within individual groups comparing rinsing and non-rinsing groups.

## Results

**Fig. 1:**
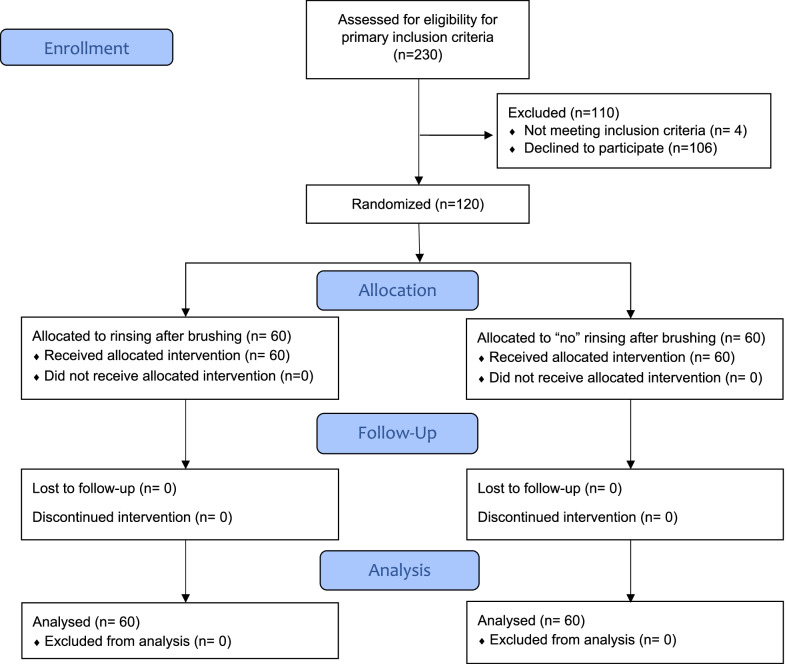
Flowchart showing the selection of the study population

Of 230 individuals considered for participation, 124 were invited to attend the screening visit. Four subjects were excluded because they did not meet the inclusion criteria. In total, 120 participants completed the study: 10 in each of the 12 study groups. Figure 1 represents the Consolidated Standards of Reporting Trials (CONSORT) flow diagram of this randomised trial.

Baseline demographics for the participants in both study groups are presented in Table 1. Age of participants ranged between 18–60 years (mean = 27.25 yrs., SD = 7.64 yrs.) with no statistically significant interaction found between the age of participants and the fluoride concentration at baseline (F = 0.97, *P* = 0.52, partial Eta squared = 0.21). The majority (66%) of participants were females. Salivary fluoride concentrations at baseline were not statistically significantly different between females and males (mean difference = 0.20 ppmF, SE = 0.52, *P* = 0.71).

**Table 1:** Baseline demographic and clinical characteristics for each group

Variable	Non-rinsing group	Rinsing group	Total	*P*
Sex				
Female (%)	42 (70)	37 (62)	79 (66)	0.22
Male (%)	18 (30)	23 (38)	41 (34)	
Age				
Range in years	18–58	18–60	18–60	0.96
Mean in years (SE)	27.22 (1.03)	27.28 (0.95)	27.25 (0.70)	
Caries Experience (mean)				
DMFT (SD)	4.78 (5.47)	4.70 (4.91)	4.74 (5.18)	0.93
DT (SD)	0.60 (2.29)	0.93 (1.73)	0.77 (2.03)	0.37
MT (SD)	0.22 (1.18)	0.12 (0.05)	0.17 (0.08)	0.53
FT (SD)	3.97 (4.19)	3.65 (3.75)	3.81 (3.97)	0.66
DMFS (SD)	8.48 (16.89)	7.62 (9.52)	8.05 (16.66)	0.73
DS (SD)	0.73 (3.17)	1.08 (2.23)	0.91 (2.74)	0.49
MS (SD)	1.05 (5.65)	0.57 (1.83)	0.81 (4.19)	0.53
FS (SD)	6.70 (11.31)	5.97 (7.55)	6.33 (9.58)	0.68
Calculus				
No (%)	43 (72)	44 (73)	87 (72.5)	0.50
Yes (%)	17 (28)	16 (28)	33 (27.5)	

For all participants, 85 (71%) did not have clinically visible caries. Salivary fluoride concentrations at baseline in caries-free participants were not significantly different from caries-active participants (mean difference = 0.39 ppmF, SE = 0.54, *P* = 0.47). No significant interaction was found between DMFT scores and salivary fluoride concentrations (F = 0.42, *P* = 0.42, partial Eta squared = 0.07). No significant interaction was found between DT scores and salivary fluoride concentrations (F = 1.01, *P* = 0.42, partial Eta squared = 0.05); between MT scores and salivary fluoride concentrations (F = 0.42, *P* = 0.74, partial Eta squared = 0.01); or FT scores and salivary fluoride concentrations (F = 0.42, *P* = 0.44, partial Eta squared = 0.06). Also, no significant interaction was found between DMFS scores and salivary fluoride concentrations (F = 0.55, *P* = 0.96, partial Eta squared = 0.14). No significant interaction was found between DS scores and salivary fluoride concentrations (F = 0.72, *P* = 0.66, partial Eta squared = 0.05); MS scores and salivary fluoride concentrations (F = 0.33, *P* = 0.86, partial Eta squared = 0.01); or FS scores and salivary fluoride concentrations (F = 0.76, *P* = 0.77, partial Eta squared = 0.16).

For all the participants, 87 (73%) did not have clinically visible calculus. The salivary fluoride concentration at baseline was not statistically significant between participants with calculus and those without calculus (mean difference = 0.08 ppmF, SE = 0.56, *P* = 0.89).

There were four missing values of salivary fluoride concentration of two participants due to technical and/or human error. The data did not follow a normal distribution and significant outliers were noticed across several time intervals. Mauchly's test of sphericity indicated that the assumption of sphericity was violated for the two-way interaction, approximate chi-squared value = 2635.75 (*P* < 0.0005). Therefore, estimates from Greenhouse–Geisser corrections were used to assess the interaction between the time and the group. There was a statistically significant two-way interaction between the time and the group on the salivary fluoride concentration (F(11.16–109.54) = 11.70, *P* < 0.0005, partial Eta squared = 0.54). This meant that the salivary fluoride concentration changed significantly over time depending on which group the participants were in.

There was no statistically significant difference in salivary fluoride concentrations between the non-rinsing groups at baseline (F = 2.07, *P* = 0.08, partial Eta squared = 0.16). There was a statistically significant effect of time on salivary fluoride concentrations for all non-rinsing groups (*P* < 0.0005) except for the control group (*P* = 0.12).

There was no statistically significant difference in salivary fluoride concentrations between the rinsing groups at baseline (F = 1.59, *P* = 0.18, partial Eta squared = 0.13). There was a statistically significant effect of time on salivary fluoride concentrations for all rinsing groups (*P* < 0.0005).

Table 2 demonstrates the mean fluoride concentration (ppmF) for all fluoride toothpaste formulas at different time intervals between rinsing and non-rinsing groups.

**Table 2:** Comparisons between the mean fluoride concentration (ppmF) at different time intervals between rinsing and non-rinsing groups

Group	Rinsing Status	Mean fluoride concentration (SD) at each study interval					
		Baseline	1 min	15 min	30 min	60 min	90 min
Control (fluoride-free)	NR	0.106 (0.154)	0.032 (0.193)	0.050 (0.071)	0.041 (0.053)	0.039 (0.060)	0.041 (0.056)
	R	0.129 (0.120)	0.037 (0.038)	0.039 (0.049)	0.023 (0.024)	0.031 (0.048)	0.020 (0.030)
	F-test	0.135	0.120	0.178	0.892	0.133	1.085
	*P*-value	0.718	0.733	0.678	0.357	0.720	0.311
Amine Fluoride (AmF)	NR	0.173 (0.205)	33.760 (17.507)	2.784 (2.214)	1.216 (1.044)	0.500 (0.365)	0.324 (0.221)
	R	0.059 (0.058)	16.865 (9.286)	1.650 (1.169)	0.561 (0.414)	0.312 (0.295)	0.174 (0.160)
	F-test	2.900	7.268	3.395	3.133	1.614	3.040
	*P*-value	0.106	0.015*	0.082	0.169	0.220	0.098
Sodium Fluoride (NaF)	NR	0.048 (0.025)	35.500 (18.351)	3.322 (2.504)	0.787 (0.523)	0.299 (0.229)	0.158 (0.095)
	R	0.063 (0.043)	15.104 (9.497)	1.701 (0.856)	0.452 (0.210)	0.213 (0.104)	0.138 (0.096)
	F-test	0.969	9.743	3.748	3.546	1.159	0.206
	*P*-value	0.338	0.006*	0.069	0.076	0.296	0.655
Sodium Monofluorophosphate (Na_2_FPO_3_)	NR	0.172 (0.143)	12.775 (4.871)	1.905 (1.281)	0.537 (0.371)	0.180 (0.101)	0.113 (0.062)
	R	0.046 (0.043)	8.976 (4.519)	0.867 (0.578)	0.260 (0.137)	0.107 (0.058)	0.058 (0.029)
	F-test	7.109	3.269	5.461	4.896	3.961	6.705
	*P*-value	0.016*	0.087	0.031*	0.040*	0.062	0.019*
Sodium Fluoride and Sodium Monofluorophosphate (NaF & Na_2_FPO_3_)	NR	0.046 (0.023)	18.118 (10.066)	1.512 (1.452)	0.369 (0.292)	0.149 (0.104)	0.105 (0.086)
	R	0.078 (0.080)	12.285 (6.486)	1.356 (0.849)	0.443 (0.459)	0.186 (0.259)	0.100 (0.120)
	F-test	1.395	2.373	0.086	0.184	0.175	0.016
	*P*-value	0.253	0.141	0.773	0.673	0.681	0.900
Stannous Fluoride and Sodium Fluoride (SnF_2_ & NaF)	NR	0.153 (0.117)	21.919 (11.677)	1.054 (0.673)	0.272 (0.154)	0.116 (0.058)	0.071 (0.034)
	R	0.087 (0.068)	17.710 (9.433)	2.245 (1.800)	0.506 (0.342)	0.175 (0.121)	0.078 (0.042)
	F-test	2.368	0.786	3.844	4.122	1.935	0.195
	*P*-value	0.141	0.387	0.066	0.057	0.181	0.664

NR, non rinsing; R, rinsing

*Statistically significant (*P* ≤ 0.05)

The only two formulas that showed a statistically significant difference at one minute between the rinsing and non-rinsing groups were AmF and NaF containing toothpaste. For Na_2_FPO_3_, statistically significantly higher fluoride concentrations were seen in the non-rinsing groups at baseline, 15, 30, and 90 min.

Comparisons between the different fluoride toothpaste formulas for the non-rinsing and rinsing groups over three different time intervals (1, 15, and 30 min) are shown in Tables 3, 4, 5, 6, 7 and 8. Na_2_FPO_3_ was the only fluoride toothpaste formula that demonstrated no statistically significant difference compared to the control group (fluoride-free toothpaste) at all time intervals for both the non-rinsing and rinsing groups. In the non-rinsing arm of the study, the highest statistically significant mean differences of fluoride concentration were reported for the NaF and the AmF groups compared to the control group at one-minute post-brushing (35.47 and 33.73 ppmF respectively).

**Table 3:** Mean differences (row-column) of fluoride concentration between toothpastes at one minute after brushing and without rinsing

Toothpaste	Control	AmF	NaF	Na_2_FPO_3_	NaF & Na_2_FPO_3_
AmF	33.73*				
NaF	35.47*	1.74			
Na_2_FPO_3_	12.74	− 20.99*	− 22.73*		
NaF & Na_2_FPO_3_	18.10*	− 15.64*	− 17.38*	5.34	
SnF_2_ & NaF	21.89*	− 11.84	− 13.58	9.14	3.80

*Statistically significant (*P* ≤ 0.05)

**Table 4:** Mean differences (row-column) of fluoride concentration between toothpastes at one minute after brushing and with rinsing

Toothpaste	Control	AmF	NaF	Na_2_FPO_3_	NaF & Na_2_FPO_3_
AmF	16.83*				
NaF	15.07*	− 1.76			
Na_2_FPO_3_	8.94	− 7.90	− 6.13		
NaF & Na_2_FPO_3_	12.25*	− 4.58	− 2.82	3.31	
SnF_2_ & NaF	17.67*	0.85	2.61	8.73	5.43

^*^Statistically significant (*P* ≤ 0.05)

**Table 5:** Mean differences (row-column) of fluoride concentration between toothpastes at 15 min after brushing and without rinsing

Toothpaste	Control	AmF	NaF	Na_2_FPO_3_	NaF & Na_2_FPO_3_
AmF	2.73*				
NaF	3.27*	0.54			
Na_2_FPO_3_	1.85	− 0.88	− 1.42		
NaF & Na_2_FPO_3_	1.46	− 1.27	− 1.81	− 0.39	
SnF_2_ & NaF	1.00	− 1.73	− 2.27*	− 0.85	− 0.46

^*^Statistically significant (*P* ≤ 0.05)

**Table 6:** Mean differences (row-column) of fluoride concentration between toothpastes at 15 min after brushing and with rinsing

Toothpaste	Control	AmF	NaF	Na_2_FPO_3_	NaF & Na_2_FPO_3_
AmF	1.61*				
NaF	1.66*	0.05			
Na_2_FPO_3_	0.83	− 0.78	− 0.83		
NaF & Na_2_FPO_3_	1.32	− 0.29	− 0.35	0.49	
SnF_2_ & NaF	2.21*	0.60	0.54	1.38*	0.89

*Statistically significant (*P* ≤ 0.05)

**Table 7:** Mean differences (row-column) of fluoride concentration between toothpastes at 30 min after brushing and without rinsing

Toothpaste	Control	AmF	NaF	Na_2_FPO_3_	NaF & Na_2_FPO_3_
AmF	1.81*				
NaF	0.75*	− 0.43			
Na_2_FPO_3_	0.50	− 0.68	− 0.25		
NaF & Na_2_FPO_3_	0.33	− 0.85*	− 0.42	− 0.17	
SnF_2_ & NaF	0.23	− 0.94*	− 0.52	− 0.27	− 0.10

^*^Statistically significant (*P* ≤ 0.05)

**Table 8:** Mean differences (row-column) of fluoride concentration between toothpastes at 30 min after brushing and with rinsing

Toothpaste	Control	AmF	NaF	Na_2_FPO_3_	NaF & Na_2_FPO_3_
AmF	0.54*				
NaF	0.43*	− 0.10			
Na_2_FPO_3_	0.24	− 0.30	− 0.19		
NaF & Na_2_FPO_3_	0.42*	− 0.12	− 0.01	0.18	
SnF_2_ & NaF	0.49*	− 0.05	0.06	0.26	0.07

^*^Statistically significant (*P* ≤ 0.05)

At 60 min, the mean difference of fluoride ranged between 0.01–0.28 ppmF for all group comparisons in the rinsing groups and 0.03–0.46 ppmF for all group comparisons in the non-rinsing groups. At this time interval, only the AmF containing toothpaste showed a significantly higher fluoride concentration (mean difference = 0.28 ppmF) compared to the control group in the rinsing arm of the study (*P* < 0.05). On the other hand, compared to the control, Na_2_FPO_3_, NaF & Na_2_FPO_3_, and SnF_2_ & NaF groups, the AmF group demonstrated a statistically significant higher saliva fluoride retention (mean difference = 0.32–0.38 ppmF) in the non-rinsing arm of the study (*P* < 0.05). All other between-group comparisons were not significant.

**Fig. 2:**
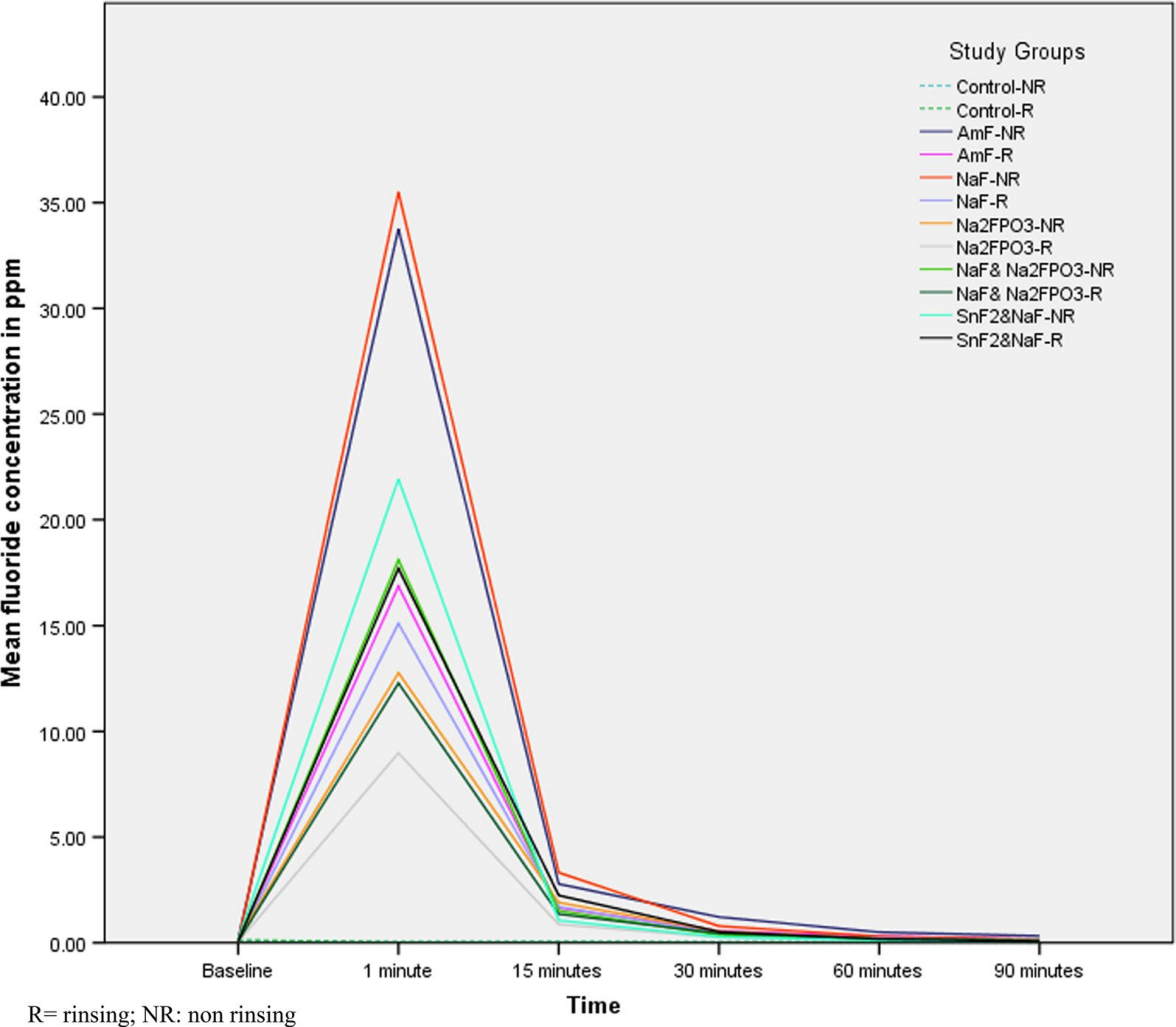
Estimated marginal means of salivary fluoride concentrations (ppmF) for 12 groups at different time intervals with and without post-brushing rinsing. R = rinsing; NR: non rinsing

At 90 min, the mean differences in fluoride concentrations ranged between 0.02–0.15 ppmF for all group comparisons in the rinsing arm of the study, and 0.01–0.28 ppmF for all group comparisons in the non-rinsing arm of the study. At this time interval, compared to the control group in both study arms, only AmF containing toothpaste showed statistically significantly higher concentrations (mean difference = 0.15 and 0.28 ppmF for the rinsing and non-rinsing groups, respectively) (*P* < 0.05). All other between-groups comparisons were not significant. Comparisons between the 12 different groups at different time intervals are presented in Fig. 2.

## Discussion

Several high-quality review articles demonstrated the role of topical fluoride treatments in significantly reducing the development of new carious lesions [19]. Strong evidence was found associating the daily use of fluoride toothpaste to a significant reduction of caries in children [19]. However, the preventive benefits of fluoride toothpaste are seldom studied in adults who have a different oral environment due to the number of restorations and missing teeth, toothbrushing routines, and other salivary factors [20]. To our knowledge, this is the first double-blinded, randomised controlled study investigating salivary fluoride concentrations after brushing with five different toothpaste formulations with two post-brushing instructions in an adult population.

To combat caries, fluoride toothpaste should result in significantly elevated and sustained levels of fluoride in saliva, and the liquid and solid phases of the dental biofilm [21]. Interest in post-brushing routines as a potential determinant of fluoride levels in saliva and therefore the anti-caries effect of fluoride toothpaste unfolded as the understanding of the topical mechanism of action of fluoride grew and the importance of oral fluoride retention became a significant factor [22]. Using toothpaste with 1450 ppmF, the fluoride concentration in saliva was found to be 100 ppmF during tooth brushing, which dropped to less than 50 ppm just after tooth brushing [23]. It was also observed that after brushing with fluoride toothpaste, salivary fluoride concentrations decreased in two distinct phases: an initial phase lasting 40–80 min and a second slow phase lasting several hours [17]. Naumova et al. [24] reported that the peak increase in salivary fluoride concentration immediately after brushing with NaF or AmF toothpaste lasted for 30 min and dropped to the baseline levels after six hours. In the current study, salivary fluoride concentration followed a similar pattern. The highest salivary fluoride concentration for all study groups was reported at one-minute post-brushing (8.98–35.5 ppmF), which dropped to 0.06–0.32 ppmF at 90 min.

There is evidence in the literature that thorough rinsing after toothbrushing accelerates the elimination of fluoride from the oral cavity [13, 17, 25]. Post-brushing rinsing was found to significantly lower salivary fluoride concentrations when compared to non-rinsing groups [12, 13, 16, 19]. On the other hand, a review by Twetman [19] concluded that evidence regarding the post-brushing practices was poor and conclusions could not be drawn. Still, current guidelines discourage post-brushing rinsing, as this practice washes away the fluoride and reduces the caries preventive effect of the fluoridated toothpaste [18, 26]. In the present study, a significant difference in salivary fluoride concentration between the rinsing and non-rinsing groups was only found in two groups (NaF and AmF) at one minute and most of the time intervals for the Na_2_FPO_3_ group. All other time intervals for most toothpaste formulas showed no significant difference between the rinsing and non-rinsing groups. It has been shown that fluoride retention in the oral cavity is influenced by many factors, such as saliva clearance, fluoride concentration of toothpaste, amount of toothpaste, and water rinsing [27, 28]. In the current study, factors like toothpaste formula, amount of toothpaste, and rinsing time and methods were standardised among the groups. However, the effects of factors like salivary clearance and fluoride reservoirs in the oral cavity of the participants were not accounted for.

Currently, there are multiple different fluoride formulations available on the market. No evidence was found relating a specific chemical formulation to caries prevention [18]. Bruun et al. [29] was one of the earliest studies that compared the concentrations of salivary fluoride after toothbrushing with NaF (500, 1000, and 1500 ppmF), and Na_2_FPO_3_ (500 and 1000 ppmF) toothpaste. The study claimed that the Na_2_FPO_3_ compound was subjected to rapid hydrolysis by bacterial phosphatase enzymes in saliva, which led to the rapid increase of fluoride ion concentration 10 min post-brushing with Na_2_FPO_3_ [29]. This was supported by trials that compared salivary fluoride levels post-brushing between NaF toothpaste (1500 ppmF) and Na_2_FPO_3_ toothpaste (1500 ppmF) [9, 17]. The study concluded that NaF toothpaste resulted in significantly higher fluoride retention when compared to Na_2_FPO_3_ toothpaste [9]. This is in agreement with the results reported from the present study, where salivary fluoride concentrations for the Na_2_FPO_3_ formula showed a high drop at 15 min in the rinsing and non-rinsing groups and without significant differences compared to the control group at any time interval. However, the combined NaF and Na_2_FPO_3_ toothpaste showed a significant increase in salivary fluoride concentration over an extended period compared to the Na_2_FPO_3_ group but not compared to the NaF only toothpaste group.

It has been shown that AmF toothpaste resulted in higher salivary fluoride concentrations compared to NaF and Na_2_FPO_3_ [11–13]. Issa and Toumba [13] conducted a randomised controlled trial to compare the salivary fluoride retention in vivo following brushing with different fluoride formulations and concentrations with and without water rinsing. They concluded that AmF toothpaste (1400 ppmF) resulted in the highest fluoride content of saliva without rinsing at 120 min. The salivary fluoride content of AmF and NaF were still higher than baseline levels after 120 min. The study did not express the results in terms of the difference in means but rather higher and lower fluoride concentration levels which makes it difficult to interpret whether any increase was likely to have influenced caries prevention. The present study agrees that AmF resulted in significantly higher salivary fluoride concentration for the longest period (90 min), for both rinsing and non-rinsing groups when compared to the control groups. This could be explained by the alignment of AmF as the hydrophilic part is arranged closely to the enamel of the tooth, while the hydrophobic part is arranged on the outside [30].

Published clinical and laboratory studies demonstrate the efficacy of SnF_2_ in reducing bacterial growth and activity, as well as protection against plaque, gingivitis, and caries [31]. However, to date, no study has investigated the saliva clearance of fluoride from SnF_2_ containing toothpaste. In the current work, a toothpaste with a combination of SnF_2_ and NaF was used. In the non-rinsing group, there was a significant difference in salivary fluoride content compared to the control group only after one minute of toothbrushing. However, in the rinsing groups, the salivary fluoride retention was higher for the SnF_2_ and NaF group compared to the control group in the first 30 min after toothbrushing.

Finally, the increase in fluoride in the oral cavity does not need to be substantial to have an anti-caries effect: even a relatively small increase in fluoride levels (from 0.03 ppm to 0.11 ppm) have been shown to enhance remineralisation, inhibit demineralisation of enamel and dentine, and reduce caries in the permanent dentition [32, 33].

This study is not without limitations. Firstly, to control the amount of water used for rinsing, the participants were asked to use a beaker of 10 ml water to rinse after toothbrushing. An earlier study found higher caries increments in participants who used a beaker of water compared to those who used other methods for rinsing after toothbrushing [15]. Secondly, when comparing the different toothpaste products and formulas, this study did not consider other product-related factors including the compatibility of active and other agents in the toothpaste which could affect the substantivity of fluoride in the oral cavity [22]. Thirdly, Duckworth and Morgan [17] found that after brushing with fluoride toothpaste, salivary fluoride can be released in a slow phase that can last for several hours. They believed that this was due to fluoride released from an oral fluoride reservoir. In the current study, this was evident where at the beginning of the study, participants in all groups had fluoride at baseline ranging between 0.05 and 0.17 ppmF. It is possible that the results of this study might have been influenced by oral fluoride reservoirs.

## Conclusions

Sodium monofluorophosphate containing toothpaste was the only formula that showed statistically significantly higher levels of fluoride in the non-rinsing group at 15-, 30- and 90-min time intervals compared to the rinsing group.

Compared to the control group, all fluoridated toothpastes were associated with higher salivary fluoride concentrations at the one-minute time interval, except for Na_2_FPO_3_ toothpaste.

AmF containing toothpaste was the only formula that showed statistically significantly higher concentrations of salivary fluoride at 90 min in both the rinsing and non-rinsing groups.

## Data Availability

All data generated or analysed during this study are included in this article. Further enquiries can be directed to the corresponding author.

## References

[1] Marinho VC, Higgins JP, Sheiham A, Logan S. Fluoride toothpastes for preventing dental caries in children and adolescents. Cochrane Database Syst Rev. 2003;(1):CD002278. 10.1002/14651858.CD002278 PMC8439270 12535435

[2] Marinho VCC (2009). Cochrane reviews of randomized trials of fluoride therapies for preventing dental caries. Eur Arch Paediatr Dent.

[3] Levine RS (2019). What concentration of fluoride toothpaste should dental teams be recommending?. Evid Based Dent.

[4] Khan IM, Mani SA, Doss JG, Danaee M, Kong LYL (2021). Pre-schoolers' tooth brushing behaviour and association with their oral health: a cross sectional study. BMC Oral Health.

[5] Twetman S (2003). Caries-preventive effect of fluoride toothpaste: a systematic review. Acta Odontol Scand.

[6] Smaïl-Faugeron V, Fron-Chabouis H, Courson F (2014). Methodological quality and implications for practice of systematic Cochrane reviews in pediatric oral health: a critical assessment. BMC Oral Health.

[7] Walsh T, Worthington HV, Glenny AM, Marinho VC, Jeroncic A (2019). Fluoride toothpastes of different concentrations for preventing dental caries. Cochrane Database Syst Rev.

[8] Beiswanger BB, Gish CW, Mallatt ME (1981). A three-year study of the effect of a sodium fluoride-silica abrasive dentifrice on dental caries. Pharmacol Ther Dent.

[9] Hirose M (2015). Fluoride retention in saliva following toothbrushing using different types of fluoridated dentifrices containing 1500 ppmF of NaF and MFP. Ped Dent J.

[10] Creeth JE (2020). In situ efficacy of an experimental toothpaste on enamel rehardening and prevention of demineralisation: a randomised, controlled trial. BMC Oral Health.

[11] Attin T, Hellwig E (1996). Salivary fluoride content after toothbrushing with a sodium fluoride and an amine fluoride dentifrice followed by different mouthrinsing procedures. J Clin Dent.

[12] Campus G, Lallai MR, Carboni R (2003). Fluoride concentration in saliva after use of oral hygiene products. Caries Res.

[13] Issa AI, Toumba KJ (2004). Oral fluoride retention in saliva following toothbrushing with child and adult dentifrices with and without water rinsing. Caries Res.

[14] Arnold WH, Dorow A, Langenhorst S, Gintner Z, Bánóczy J, Gaengler P (2006). Effect of fluoride toothpastes on enamel demineralization. BMC Oral Health.

[15] Chestnutt IG, Schafer F, Jacobson AP, Stephen KW (1998). The influence of toothbrushing frequency and post-brushing rinsing on caries experience in a caries clinical trial. Community Dent Oral Epidemiol.

[16] Nazzal H, Duggal MS, Kowash MB, Kang J, Toumba KJ (2016). Comparison of residual salivary fluoride retention using amine fluoride toothpastes in caries-free and caries-prone children. Eur Arch Paediatr Dent.

[17] Duckworth RM, Morgan SN (1991). Oral fluoride retention after use of fluoride dentifrices. Caries Res.

[18] Scottish Intercollegiate Guidelines Network. 2014. Dental Interventions to Prevent Caries in Children: A national clinical guideline. [SIGN 138]. [Online] Edinburgh: Scottish Intercollegiate Guidelines Network. https://www.scottishdental.org/wp-content/uploads/2014/04/SIGN138.pdf. Accessed 12 Oct 2021.

[19] Twetman S (2009). Caries prevention with fluoride toothpaste in children: an update. Eur Arch Paediatr Dent.

[20] Reich E (2001). How to measure the effects of fluoride treatments in clinical trials? The role of caries prevalence and caries assessment. Caries Res.

[21] Twetman S (2018). Prevention of dental caries as a non-communicable disease. Eur J Oral Sci.

[22] Parnell C, O'Mullane D (2013). After-brush rinsing protocols, frequency of toothpaste use: fluoride and other active ingredients. Monogr Oral Sci.

[23] Ekstrand KR (2016). High fluoride dentifrices for elderly and vulnerable adults: does it work and if so, then why?. Caries Res.

[24] Naumova EA (2012). Fluoride bioavailability in saliva and plaque. BMC Oral Health.

[25] Tenuta LMA, Cury JA (2013). Laboratory and human studies to estimate anticaries efficacy of fluoride toothpastes. Monogr Oral Sci.

[26] Toumba KJ (2019). Guidelines on the use of fluoride for caries prevention in children: an updated EAPD policy document. Eur Arch Paediatr Dent.

[27] Mystikos C, Yoshino T, Ramberg P, Birkhed D (2011). Effect of post-brushing mouthrinse solutions on salivary fluoride retention. Swed Dent J.

[28] Creeth J, Zero D, Mau M, Bosma ML, Butler A (2013). The effect of dentifrice quantity and toothbrushing behaviour on oral delivery and retention of fluoride in vivo. Int Dent J.

[29] Bruun C, Givskov H, Thylstrup A (1984). Whole saliva fluoride after toothbrushing with NaF and MFP dentifrices with different F concentrations. Caries Res.

[30] Priyadarshini Sh, Raghu R, Shetty A, Gautham P, Reddy S, Srinivasan R (2013). Effect of organic versus inorganic fluoride on enamel microhardness: an in vitro study. J Conserv Dent.

[31] Sensabaugh C, Sagel ME (2009). Stannous fluoride dentifrice with sodium hexametaphosphate: review of laboratory, clinical and practice-based data. J Dent Hyg.

[32] ten Cate JM, Featherstone JD (1991). Mechanistic aspects of the interactions between fluoride and dental enamel. Crit Rev Oral Biol Med.

[33] Toumba KJ, Curzon ME (2005). A clinical trial of a slow-releasing fluoride device in children. Caries Res.

